# Serum antioxidant micromineral (Cu, Zn, Fe) status of drug dependent subjects: Influence of illicit drugs and lifestyle

**DOI:** 10.1186/1747-597X-2-12

**Published:** 2007-04-08

**Authors:** Kazi Jahangir Hossain, Md Mustafa Kamal, Monira Ahsan, SK Nazrul Islam

**Affiliations:** 1 Institute of Nutrition and Food Science, University of Dhaka, Dhaka-1000, Bangladesh; 2 Department of Pharmaceutical Chemistry, University of Dhaka, Dhaka-1000, Bangladesh

## Abstract

**Background:**

Use of illicit drugs induces multiple nutrient deficiencies. Drug habit, sexual practice and socioeconomic factors influence the nutrient profile of drug dependent subjects. The literature on this issue is still insufficient. This study has tested the hypothesis that illicit drug use and lifestyle impair mineral status. To test this hypothesis, 253 men multiple drug users of age 18–45 years were recruited to investigate their serum copper, zinc and iron levels. Influence of illicit drugs and their lifestyle on the mineral levels was also examined. The study subjects were drug dependent who had shared needles and had sexual activity with multiple partners. Serum concentrations of the minerals were estimated by atomic absorption flame spectrometry.

**Results:**

Results showed a significant increase in serum copper and zinc concentrations, and decrease in iron level in drug dependent subjects. The increase of copper level was found to be much higher than that of zinc. Period of drug abuse had made a significant positive influence on the copper and iron levels, but it was apparently reversed for zinc concentration. Multiple sexual partnerships had significant influence on zinc status. There also were significant relationships observed between body mass index (BMI) as well as certain socioeconomic factors, and mineral status of drug dependent subjects and non-drug dependent controls. A series of multiple linear regression analysis predicted mineral values for education, age and BMI. The group (drug dependent subject = 1, non-drug dependent control = 2) had a significant influence on these parameters. However, after controlling these factors, it was shown that illicit drug use significantly contributed to influence the serum mineral levels.

**Conclusion:**

Illicit drug use impairs serum mineral value causing an increase in copper and zinc and a decrease in iron. Lifestyle and nutritional status of drug dependent subjects influence serum mineral concentrations.

## Background

It has been documented that use of illicit drugs induces multiple nutrient deficiencies or malnutrition [1,2], which is the most common cause of immunodeficiency [3-6]. Immunocompetence is a sensitive and functional determinant of nutritional status because it is altered even before the onset of clinical symptoms of malnutrition [1]. Illicit drugs are themselves immunosuppressive [7-11] and the use of these drugs undermines appetite [12] and affects food habits [13] making those who are drug dependent crave empty-calorie nutrient deficient foods [2,14,15]. This may cause micronutrient deficiencies, and thus influences susceptibility to infectious agents including HIV infection [1,2]. In addition, behavioral risk factors in drug dependent subjects such as sexual practice, unprotected sex with multiple partners, needle sharing etc [16-18] also ranks those who are drug dependents to be at the highest risk of HIV infection [2,19].

Microminerals or trace elements play a versatile function in human body ranging from developing immunity to provide antioxidant defense [20-25]. Zinc is essential for its catalytic, structural and regulatory functions. Its metalloenzymes are involved in immune development, cognitive functioning, reproductive maturation and physiological growth [20,21]. Zinc is required for DNA replication, RNA transcription, cell division and cell activation. Copper and iron are also crucial for physiological functions, antioxidant defense, and immune development [23,25-27]. Deficiency of any of these elements badly affects normal functions in the human body. It is also further reported that overload of micromineral or trace elements produces immunotoxicity [3,4]. Currently some investigators have been reporting a change in serum trace element contents in drug dependent individuals [28-30]. In view of their potential immunonutritional functions, we report here serum copper, zinc and iron status of drug dependent subjects who were being dependent to multiple immunosuppressive illicit drugs.

## Results

This study has investigated two hundred fifty-three male multiple illicit drug users of age 18–45 years. Their serum micromineral concentrations were analysed and compared with non-drug dependent control subjects. Influence of illicit drugs and lifestyle of drug dependent subjects on their serum mineral values was also addressed.

Table 1 shows a significant change in serum micromineral concentrations in drug dependent subjects. Concentrations of copper, zinc and iron in the drug dependent subjects were 21.6 ± 5.8 μmol/L, 13.8 ± 4.5 μmol/L and 20.5 ± 6.5 μmol/L, while the concentrations were 15.2 ± 4.1 μmol/L, 12.3 ± 4.1 μmol/L and 32.4 ± 8.9 μmol/L in the non-drug dependent controls, respectively. These values indicated a significant increase in serum copper and zinc concentrations and a decrease in iron concentration in the drug dependent subjects. The increase of copper level was found to be much higher than that of the zinc.

**Table 1:** Serum micromineral levels in drug dependent subjects (n = 253) and non-drug dependent controls (n = 100).

Micromineral (μmol/L)	Drug addict			Non-addict control			P-value*
		
	N	%	Mean ± SD	N	%	Mean ± SD	
Copper							
9.0–10.9	7	2.8	21.6 ± 5.8	20	20.0	15.2 ± 4.1	t = 10.001
11.0–21.9	132	52.1		74	74.0		p = 0.002
22.0–45.0	114	45.1		6	6.0		
Zinc							
5.0–11.5	80	31.6	13.8 ± 4.5	53	53.0	12.3 ± 4.1	t = 3.00
11.6–18.5	137	54.2		39	39.0		p = 0.001
18.6–40.0	36	14.2		8	8.0		
Iron							
8.5–14.5	34	13.4	20.5 ± 6.5	0		32.4 ± 8.9	t = 13.94
14.6–32.2	206	81.4		48	48.0		p = 0.001
32.3–42.0	13	5.2		52	52.0		

*Significant (p < 0.05).

Legend: Replicate analyses were carried out for every sample.

Descriptive statistics: Frequencies, descriptive, crosstabs.

Compare means: Independent-sample t-test.

One-way analysis of variance showed an nonsignificant trend of increasing copper and iron with multiple drug use (table 2). Period of drug abuse showed a positive relation with copper [F(4,250) = 3.45, p = 0.03] and iron [F(4,250) = 5.20, p = 0.001]; that is, a longer period of drug abuse induced a greater increase of copper and iron levels. Serum zinc level was not influence by period of drug abuse. Zinc was found to be influenced by multiple sexual partners [F(3,249) = 4.84, p = 0.001]. Condom use or STIs (sexually transmitted infections) did not show any particular influence on the microminerals.

**Table 2:** Effect of drug habit and sex practice on micromineral status (mean ± sd in μmol/L) of drug dependent subjects.

Parameter	% (n)	Copper^a ^(μmol/L) Mean ± SD	Zinc^b ^(μmol/L) Mean ± SD	Iron^c ^(μmol/L) Mean ± SD
**Number of drugs**^1^				
1–2	39.9 (101)	21.3 ± 5.8	13.8 ± 4.5	20.0 ± 5.9
3	37.9 (96)	21.2 ± 5.7	14.1 ± 5.0	20.3 ± 6.9
4–5	22.2 (56)	22.6 ± 6.2	13.5 ± 3.6	22.1 ± 7.3
**Period of drug abuse**^2^				
1–5 y	38.7 (98)	20.9 ± 5.2*	14.5 ± 5.0	19.9 ± 5.9*
6–10 y	42.7 (108)	21.4 ± 5.8 *	13.5 ± 3.8	20.0 ± 7.1*
11–40 y	18.6 (47)	23.5 ± 6.8*	13.1 ± 4.9	23.3 ± 5.8*
**Sex partner**^3^				
0–5	37.5 (95)	20.1 ± 5.0	13.8 ± 4.2*	19.4 ± 5.9
6–10	20.2 (51)	22.2 ± 6.6	15.8 ± 6.4*	20.9 ± 7.2
11–15	12.3 (31)	22.3 ± 7.2	12.6 ± 3.5*	22.6 ± 6.1
16–150	30.0 (76)	21.5 ± 5.7	13.1 ± 3.4*	20.7 ± 6.8
**Condom use**^4^				
Occasional use	23.7 (60)	21.9 ± 6.9	13.3 ± 4.6	20.8 ± 61
No use	76.3 (193)	21.5 ± 5.5	14.0 ± 4.5	20.4 ± 6.6
**STD**^5^				
Syph-gonorrhoea	59.3 (150)	21.4 ± 6.1	13.4 ± 4.9	20.9 ± 6.5
No STD	40.7 (103)	21.9 ± 5.5	14.5 ± 3.9	19.9 ± 6.1

* Significant (p < 0.05) using one-way ANOVA.

Number of drugs: 1 = heroin, 2 = cannabis, 3 = phensedyl (codein, ephedrine and promethazine), 4 = injections (pethedine, buprenorphine, diazepam, promethazine), 5 = tablets (benzodiazepines).

^2a^F(2,250) = 3.45, p = 0.030

^2c^F(2,250) = 5.20, p = 0.001

^3b^F(3,249) = 4.84, p = 0.003

Serum micromineral status was influenced by some socioeconomic factors (table 3). In the drug dependent subjects, there was a significant positive relationship between education and zinc (F(1,251) = 4.44, p = 0.036), and a negative relationship between education and copper (F(1,251) = 5.01, p = 0.026). Age had a positive relationship with serum copper (F(1,251) = 8.47, p = 0.004) and a negative relationship with zinc (F(1,251) = 3.99, p = 0.047). BMI had a positive relationship with zinc (F(1,251) = 10.1, p = 0.002), and a negative relationship with copper (F(1,251) = 5.83, p = 0.017) and iron (F(1,251) = 6.59, p = 0.011). For the non-drug dependent controls, education positively influenced copper (F(1,98) = 10.0, p = 0.002) and zinc (F(1,98) = 17.4, p = 0.001). Occupation did influence all of the mineral values.

**Table 3:** Influence of socioeconomic factors and BMI on serum micromineral status of drug dependent subjects and non-drug dependent controls.

		Copper (μmol/L)^a^	Zinc (μmol/L)^b^	Iron (μmol/L)^c^
Parameter	% (n)	Mean ± SD	Mean ± SD	Mean ± SD
**Drug dependent subjects**				
Education^1^				
0–10 class	61.3(155)	22.24 ± 5.95*	13.36 ± 4.27*	20.85 ± 6.56
11–15 class	38.7(98)	20.62 ± 5.59*	14.59 ± 4.83*	19.93 ± 6.37
Occupation^2^				
Business	45.5(115)	21.82 ± 5.78	14.37 ± 4.90	20.60 ± 6.35
Others	54.5(138)	21.51 ± 5.94	13.40 ± 4.16	20.41 ± 6.62
Monthly income US$^3^				
20–100	59.7(151)	21.81 ± 6.11	13.45 ± 4.68	20.77 ± 6.73
101–890	40.3(102)	21.40 ± 5.49	14.42 ± 4.26	20.09 ± 6.12
Age in year^4^				
18–29	63.6(161)	20.95 ± 5.67*	14.27 ± 4.90*	20.36 ± 6.57
30–45	36.4(92)	23.05 ± 5.96*	13.10 ± 3.72*	20.73 ± 6.38
Marital status^5^				
Married	59.7(151)	22.13 ± 5.68	13.98 ± 4.69	21.20 ± 6.58
Unmarried	40.3(102)	20.93 ± 6.07	13.63 ± 4.29	19.45 ± 6.23
Body mass index^6^				
11.5–18.4	65.6(166)	22.29 ± 6.16*	13.20 ± 3.90*	21.24 ± 6.59*
18.5–28.5	34.4(87)	20.43 ± 5.05*	15.07 ± 5.35*	19.07 ± 6.07*

**Non-drug dependents**				
Education^7^				
0–10 class	56.0(56)	14.16 ± 3.79*	10.89 ± 3.08*	33.75 ± 8.34
11–15 class	44.0(44)	16.65 ± 4.03*	14.05 ± 4.50*	30.74 ± 9.35
Occupation^8^				
Business	43.0(44)	13.11 ± 3.37*	10.03 ± 2.33*	34.74 ± 8.10*
Others	57.0(56)	16.94 ± 3.79*	14.05 ± 4.27*	30.60 ± 9.11*
Monthly income US$^9^				
20–100	62.0(62)	15.16 ± 4.29	12.11 ± 3.91	32.35 ± 8.55
101–890	38.0(38)	15.40 ± .74	12.55 ± 4.36	32.54 ± 9.51
Age in year^10^				
18–29	59.0(59)	15.24 ± .4.15	12.75 ± .4.01	32.30 ± .8.94
30–45	41.0(41)	15.28 ± .4.01	11.61 ± .4.10	32.61 ± .8.90
Marital status^11^				
Married	55.0(55)	15.03 ± .4.14	11.19 ± .3.85*	33.16 ± .8.70
Unmarried	45.0(45)	15.53 ± .4.02	13.62 ± .3.96*	31.53 ± .9.11
Body mass index^12^				
11.5–18.4	15.0(15)	15.98 ± .3.95	11.99 ± .3.01	33.12 ± .9.20
18.5–28.5	85.0(85)	15.13 ± .4.10	12.33 ± .4.24	32.30 ± .8.87

*Significant (p < 0.05) using one-way ANOVA.

^1a^F(1,251) = 5.01, p = 0.026

^1b^F(1,251) = 4.44, p = 0.036

^4a^F(1,251) = 8.47, p = 0.004

^4b^F(1,251) = 3.99, p = 0.047

^5c^F(1,251) = 4.47, p = 0.035

^6a^F(1,251) = 5.83, p = 0.017

^6b^F(1,251) = 10.1, p = 0.002

^6c^F(1,251) = 6.59, p = 0.011

^7a^F(1,98) = 10.0, p = 0.002

^7b^F(1,98) = 17.4, p = 0.001

^8a^F(1,98) = 27.7, p = 0.001

^8b^F(1,98) = 31.6, p = 0.001

^8c^F(1,98) = 5.59, p = 0.020

^11b^F(1,98) = 9.62, p = 0.003.

The above findings indicated that illicit drug use, socioeconomic factors and BMI had a significant influence on the serum mineral status (table 1 and 3). It was therefore necessary to determine, in addition to illicit drugs, to what extent did the socioeconomic factors and BMI contribute to the serum mineral values. In order to find these relationships, a multiple linear regression model was used with copper, zinc and iron as dependent variables. First, bivariate correlations were performed to find the relationship between the dependent variables and independent socioeconomic factors and BMI of both drug dependent subjects and non-drug dependent controls (table 4). These showed a significant relationship between copper, education, age and BMI; zinc, age and BMI; and iron and marital status. Therefore, controlling these independent factors that affected the serum mineral status, a series of linear regression analysis was performed. In the first analysis, age and BMI showed a significant predicted relation with copper. After controlling the age and BMI, the groups (drug dependent subjects = group 1, non-drug dependent = group 2) also showed a significant negative influence on copper. Similarly when zinc and iron were used as dependent variables, the groups had an independent influence on each of them, even after controlling other socioeconomic variables in favor of controls (table 4).

**Table 4:** Correlation coefficient (r), Regression coefficient (β) of multiple regression analysis.

Parameter^Ψ^	Correlation coefficient (r) (Significance 2-tailed, p)	Regression coefficient (β) (Significance, p)
**Copper **(R^2 ^= 0.250)		
Education	-0.126 (0.045)	-0.070 (0.291)
Age	0.149 (0.018)	0.126 (0.044)
BMI	-0.211 (0.001)	-0.181 (0.006)
Group		-0.471 (0.001)
**Zinc **(R^2 ^= 0.076)		
Age	-0.148 (0.019)	-0.119 (0.058)
BMI	0.205 (0.001)	0.175 (0.008)
Group		-0.158 (0.003)
**Iron **(R^2 ^= 0.366)		
Marital status	-0.132 (0.035)	-0.056 (0.281)
Group		0.373 (0.001)

*Significant (p < 0.05) obtained from a t-test for regression coefficient (β)

^Ψ^Copper, zinc and iron were taken as dependable variables. The independent variables (education, age, BMI, marital status) that were significantly correlated with the dependable variables were mentioned only. Group indicated: group 1 = drug dependent subjects, group 2 = non-dependent controls.

## Discussion

Use of illicit drug or drug abuse induces a change in serum micromineral or trace element level [28-30]. Our study showed that illicit drug use produced an increase of serum copper and zinc concentrations and a decrease of iron concentration. Change in trace element level due to drug abuse has also been reported by some investigators [28-32], where they observed an increase in copper and a decrease in zinc in most of the addicts or drug dependent individuals, and in a few cases, both an increase and a decrease in iron concentration was reported. Our zinc outcome is contrary to their observations. The increase of copper level may possibly be associated with stress, inflammation and infections [33,34] and the increase of zinc may be because of acute fasting [35], all of which are reported to be prevalent in drug addicts or drug dependents [1,36]. It is further reported that acute fasting causes a release of zinc from the liver that adds up to blood zinc making a higher blood zinc level. However, it may be suggested that since zinc is potentially involved in regulating immune responses [24], the increased zinc may be utilized to up-regulate the immune function of drug dependent subjects. The decreased iron level may be associated with malnutrition [37] and vitamin-A deficiency [38] which is also prevalent in drug addicts or dependant patients [39,40].

One-way analysis of variance indicated a positive relationship between period of drug use and concentrations of copper and iron. Since the use of illicit drugs causes a rise of serum copper, the longer period of drug abuse should, therefore, result in a much higher increase in copper level. The influence of abuse period on serum iron is conflicting with our result, the reason of which is not clear to us. However, certain adaptive mechanism or damage to some mechanisms or organs may develop with longer period of drug abuse that may prevent iron utilization causing a rise of circulating iron level. There was a negative influence of multiple sexual partnerships on zinc. How this is happening is not known. However, the possible explanation may be that because of unprotected sexual act, sex with multiple sexual partners and needle sharing may increase the spreading of hepatitis, STIs, and other infections including HIV in the drug dependent subjects [16,18]; as infections are synergistic with nutritional deficiencies, these infections in drug dependent subjects may result to further overstress zinc deficiency. This is how multiple sexual partnerships may cause lowered serum zinc.

Some socioeconomic factors and BMI had an influence on serum trace elements in both drug dependent subjects and non-drug dependent controls. The positive relationship between the socioeconomic factors and trace elements is well predicted, but their negative association is unexpected. However, infection, stress and fasting [35,37], food habit [2] and multiple malnutrition [1,2] might be linked to this unpredicted outcome.

Use of illicit drugs and some socioeconomic factors as well as BMI did have influence on the serum mineral concentrations. In order to determine what extent the socioeconomic factors and BMI influence the mineral concentrations, a multiple regression analysis was performed. Results indicated that, in addition to the illicit drugs, education, age and BMI had significant influence on the serum mineral index. After controlling these factors, it was evident that illicit drug itself impaired the serum copper, zinc and iron concentrations. This outcome is consistent to the well-documented contributory role of socioeconomic factors to the nutrition of an individual.

## Conclusion

This study reveals that use of illicit drugs impairs serum mineral level causing an increase of copper and zinc and a decrease of iron. Lifestyle and nutritional status of drug dependent subjects influence serum mineral concentrations. As nutritional deficiencies or poor nutritional status induce immunodeficiency [3,4] that may influence susceptibility to HIV infection [41-44], a careful multiple micronutrient intervention would be of particularly importance in the clinical management of drug dependent patients, as well as of the HIV infected or AIDS patients.

## Methods

### Sample

This was a case-control study conducted among two hundred fifty three men of age 18–45 y, who were drug dependent. They were multiple drug users who principally used heroin, phensedyl, cannabis, injection drugs (pethedine or buprenorphine), and sought detoxification therapy voluntarily at the Central Drug Addiction Treatment Hospital, Tejgaon, Dhaka. The drug dependent subjects were recruited under defined criteria excluding medical disorders and precluded an acute or chronic illness, impaired hepatic or renal functions, cardiac disorders, tuberculosis, severe asthma, using prescribed medicines and alcohol. In addition, re-admissions within one year and a period of drug abuse less than one year were also excluded. One hundred non-drug dependent (non-users of any tobacco, betel leaves or nuts) healthy men who were similar in age, height, and socioeconomic status were recruited by convenience as controls from the same community.

Blood specimen and a pre-tested questionnaire were used as research tools. The questionnaire was design to query *drug habits *such as – number of illicit drugs taken, period of drug abuse; *sexual behaviors *such as number of sex partners (lifetime), condom use, sexually transmitted infections (STIs, gonorrhea and syphilis); *socioeconomic information *such as education, occupation, monthly income, age, marital status; and *nutritional status *such as body mass index (BMI).

The study subjects were briefed about the objective of the study and a written consent was obtained from each of them. Socioeconomic information was recorded at the time of admission into the hospital. Then blood specimen was collected from each of the subjects. To maintain privacy, a resident physician confidentially collected the information on drug habit and sexual practices of drug dependent subjects. Body weight and height were then measured using a physician adult metric scale while wearing no shoes. Ethical permission was obtained from the Central Drug Addiction Treatment Hospital.

### Blood analysis

A venous blood sample of 3–5 ml was collected from each of the participating drug dependent subjects and non-drug dependent controls. After collection, blood was kept undisturbed for at least 60 min and then centrifuged at 3000 rpm for 10 min to extract serum. Serum thus extracted was stored at -20°C for analysis of minerals.

Concentrations of copper, zinc and iron in the sera were determined by atomic absorption flame spectrophotometry as described elsewhere [45]. Pure copper, zinc and iron solutions (Sigma Chemicals Co, St. Louis, MO, USA) were used as reference standard material. Five concentrations such as 20, 40, 60, 80 and 100 μg/dl for copper and zinc, and 50, 100, 150, 200 and 250 μg/dl for iron were prepared for standard calibration graphs for the respective minerals. A software package (AA-6200, ver 1.1, Shimadzu Corporation, Kyota, Japan) was used to construct the calibration graphs. For analysis of copper and iron, serum was diluted ×10 (9:1) with de-ionized water, and for zinc, serum was diluted 10× (9:1) with de-ionized saline water (0.82% NaCl). Hemorrhagic or cloudy serum was treated with 5% TCA and the clear supernatant was used. Absorbance for copper, zinc and iron were read at 324.8 nm, 213.9 nm and 248.3 nm respectively in the atomic absorption flame spectrometer (AA-6200 Series, Shimadzu Corporation, Kyota, Japan). To maintain and verify assay accuracy, the standard mineral preparations were run for every 20 test samples. The software package (AA-6200, ver 1.1, Shimadzu Corporation) was used to calculate the concentrations of copper, zinc and iron. In order to avoid or minimize trace elements contamination, adequate precautions were taken during collection and subsequent processing of blood and serum.

### Statistical analysis

SPSS software package (version 12.0 SPSS Inc, Chicago, IL, USA) was used to analyze the data. Descriptive statistics were used for all variables. Values were expressed as percentage, mean and standard deviation. Comparison of minerals contents of drug dependent subjects to that of the non-drug dependent controls was performed by independent sample t-test. One-way analysis of variance (ANOVA) was used to assess the influence of drug habit, sexual practice, socioeconomic factors and BMI on serum copper, zinc and iron. Bivariate linear correlation and multiple regression analyses were performed to determine the extent of contribution of socioeconomic factors and BMI to influence the serum minerals concentrations.

## Competing interests

The author(s) declare that they have no competing interests.

## Authors' contributions

All the authors have contributed to this research work. Kazi Jahangir Hossain, who is a physician, participated in recruiting drug dependent subjects, collection of data and blood specimens, and in carrying out laboratory and statistical analyses. Md Mustafa Kamal, who is also a physician, participated in carrying out laboratory and statistical analyses. Monira Ahsan contributed to intellectual contribution to conception and design and, to some extent, interpreting results. SK. Nazrul Islam made the major contribution to conception and design, support facilities, acquisition of funding to conduct this research work, and prepared and revised the manuscript. All authors read and approved the final manuscript.
